# The role of lake size and local phenomena for monitoring ground-fast lake ice

**DOI:** 10.1080/01431161.2018.1519281

**Published:** 2018-09-26

**Authors:** Georg Pointner, Annett Bartsch, Bruce C. Forbes, Timo Kumpula

**Affiliations:** a Austrian Polar Research Institute, Vienna, Austria; b b.geos, Korneuburg, Austria; c Zentralanstalt für Meteorologie und Geodynamik, Vienna, Austria; d Arctic Centre, University of Lapland, Rovaniemi, Finland; e Department of Geographical and Historical studies, University of Eastern Finland, Joensuu, Finland

## Abstract

In this study, we assess the effect of the lake size on the accuracy of a threshold-based classification of ground-fast and floating lake ice from Sentinel-1 Synthetic Aperture Radar (SAR) imagery. For that purpose, two new methods (flood-fill and watershed method) are introduced and the results between the three classification approaches are compared regarding different lake size classes for a study area covering most of the Yamal Peninsula in Western Siberia. The focus is on April, the stage of maximum lake ice thickness, for the years 2016 and 2017. The results indicate that the largest lakes are likely most prone to errors by the threshold classification. The newly introduced methods seem to improve classification results. The results also show differences in fractions of ground-fast lake ice between 2016 and 2017, which might reflect differences in temperatures between the winters with severe impact on wildlife and freshwater fish resources in the region. Patterns of low backscatter responsible for the classification errors in the centre of the lakes were investigated and compared to the optical Sentinel-2 imagery of late-winter. Strong similarities between some patterns in the optical and SAR data were identified. They might be zones of thin ice, but further research is required for clarification of this phenomenon and its causes.

## 1. Introduction

Lakes and ponds are ubiquitous features of the arctic tundra landscape (Smith, Sheng,and MacDonald 2007). They occur in a wide range of sizes and depths (Bartsch et al. 2017). Many of them are relatively shallow, reaching depths of only a few metres (Muster et al. 2017). A large proportion of lakes in Siberia and Alaska are associated with thermokarst phenomena (thaw lakes), meaning that they formed over long time periods (e.g. West and Plug 2008). Some thermokarst lakes are considered to be large sources of methane (CH_4_) emissions, particularly those which lie above highly organic substrates, such as yedoma silts- soils (Walter et al. 2006; Murton et al. 2015). Methane may also be emitted during the winter from those portions of the lakes that do not freeze to the ground (Wik et al. 2011). Depending on their bathymetric profiles, they maintain different fractions of liquid water during wintertime. Many lakes freeze to the ground (ground-fast or bottom-fast lake ice) only along their rims and keep liquid water under an ice layer on top (floating lake ice) in their deeper parts (Jeffries et al. 1994). Very shallow lakes freeze entirely to the ground in late winter and in larger, deeper lakes water remains liquid under floating ice nearly throughout the whole lake area. The maximum ice thickness is expected to be sensitive to climate change (Duguay et al. 2003; Jeffries, Morris, and Duguay 2005; Brown et al. 2010). In recent decades, a steady decrease in ground-fast lake ice area has been observed on the Alaskan North Slope, which is attributed to climate change-induced decline in maximum ice thickness (Surdu et al. 2014). Changes in sublake temperatures are relevant for maintenance of permafrost below (Arp et al. 2016). Mapping areas of floating lake ice, and tracking long-term changes in these areas, might be also of interest for emission studies. As lakes that maintain liquid water throughout the whole winter provide important overwintering habitats for many arctic freshwater fish species (Jones et al. 2009), information on the distribution of ground-fast lake ice can also be useful for ecological studies (e.g. Earnst 2004). Knowing freshwater availability during late winter can also be important for ice road construction, where water is usually pumped from lakes and added to the ground in layers or by flooding (White et al. 2008).

Satellite-based Synthetic Aperture Radars (SARs) provide day and night observations unaffected by cloud cover and largely independent of weather conditions. Several studies have successfully demonstrated the ability of using C-Band SAR data to distinguish between areas of ground-fast and floating lake ice of thermokarst lakes (e.g.Jeffries, Wakabayashi, and Weeks 1993; Morris, Jeffries, and Weeks 1995; Brown et al. 2010; Arp et al. 2011; Duguay et al. 2002; Bartsch et al. 2017; Engram et al. 2018). Low backscatter from ground-fast ice and high backscatter from floating ice have been well documented and explained. A range of studies which focus on permafrost applications has already been analysing ground-fast lake ice at its maximum ice thickness in late winter using SAR data (Arp et al. 2011, 2012; Grunblatt and Atwood 2014; Engram et al. 2013a; Surdu et al. 2014).

The relatively low backscatter from shallower parts of lakes can be explained by the low contrast between the real parts of the dielectric constants of the ice and the frozen soil beneath it (Duguay et al. 2002). ${\varepsilon_{r}}^{\prime }$ of freshwater ice is usually between 2.2 and 4.5, ${\varepsilon_{r}}^{\prime }$ of the lake sediments below the ice usually 3.2 to 8.0. The radar waves penetrate nearly unaffected through the ice layer and also at the ice-soil boundary, most of the signal is transmitted and absorbed by the lake sediments.

The dominant mechanism for the observed high backscatter from floating lake ice is however not as clear. The strong backscatter has long been considered to be caused by a double-bounce effect from columnar bubbles trapped within the ice (typical for shallow arctic lakes) and the ice-water boundary (e.g. Wakabayashi, Weeks, and Jeffries 1993; Jeffries et al. 1994). This model assumes that the signal penetrates through a part of the ice layer, is then scattered by bubble inclusions within the ice, where most of it is reflected specularily, and subsequently mainly reflected specularily again by the ice-water boundary because of the high dielectric contrast (${\varepsilon_{r}}^{\prime }$ of water is approximately 80). This effect would cause most of the signal to return in nearly the same direction from which it was transmitted. Recent studies using polarimetric SAR data, however, provide strong evidence that the main parameter responsible for the observed strong backscatter is the roughness at the ice–water interface (Engram et al. 2013b; Atwood et al. 2015). In this model, the strong radar return is caused by local angular differences at the ice water boundary.

While a range of studies is available focusing on characterizing and modelling backscatter from shallow frozen lakes and SAR data has been used to distinguish lakes that maintain liquid water throughout winter versus lakes that do not (Arp et al. 2011), few approaches to map the areal extent of ground-fast and floating lake ice can be found in the literature. Two classification methods can be found: Threshold-based classification and Iterative Region Growing using Semantics (IRGS). Atwood (2014) used a statistically derived threshold to map areas of floating lake ice for freshwater availability on the North Slope of Alaska from European Remote Sensing Satellite 2 (ERS-2) C-band vertical-vertical (VV) polarized SAR (75 m) scenes. An incidence angle dependent threshold backed by bathymetric data has been proposed by Bartsch et al. (2017) to enable circumpolar mapping with Environmental Satellite (ENVISAT) Advanced Synthetic Aperture Radar (ASAR) C-band horizontal-horizontal (HH) polarized observations. Scene-specific automatic threshold determination allows the combination of different polarizations and sensor generations (Engram et al. 2018). An IRGS approach was applied by Surdu et al. (2014), where the Map-Guided Ice Classification System (MAGIC) by Clausi et al. (2010) was used to classify a 20-year time series of ERS-1 and ERS-2 C-band VV-polarized imagery (240 m low resolution mode) acquired over the North Slope of Alaska in order to determine changes in the extent of ground-fast lake ice. Jeffries, Morris, and Kozlenko (2005) and Bartsch et al. (2017) discuss the occurrence of fractures and related features for larger lakes, but so far, the effect of lake size on the classification results has not been assessed for any study area.

Neither ERS nor ENVISAT remains in operation. The newest generation of European earth observation satellites are the Sentinel satellites, which are part of the Copernicus program of the European Union (EU) (Torres et al. 2012). Among them are two radar-satellites (Sentinel-1A and Sentinel-1B) equipped with identical SAR instruments (C-SAR) operating in C-band. Sentinel-1A was launched in April 2014, Sentinel-1B in April 2016. In Extra Wide swath mode (EW), backscatter measurements are acquired with a comparably high spatial resolution of approximately 40 m (Geudtner et al. 2014), similar to ERS SAR standard mode (30 m). This is a higher resolution than the data used by Bartsch et al. (2017).

It is expected that phenomena such as cracks impact the separability of ground-fast ice in this case. It is assumed that these phenomena have a greater effect for larger lakes. The aim of this study is to quantify the impact of such local phenomena for the retrieval. We consider lake size as the basis for this assessment. We tested Sentinel-1 EW data for large-scale mapping of ground-fast lake ice over a study area covering the Yamal Peninsula in Western Siberia. To account for the effect of presumably falsely classified pixels, two new methods for mapping ground-fast lake ice are introduced and compared to the results of the threshold based classification. Patterns of low backscatter in central parts of large lakes in the SAR images are compared to optical images. In addition, both winters with available data, 2016 and 2017, are compared.

## 2. Study area and data

### 2.1. Study area

The study area lies in Western Siberia, North of the Arctic Circle. By far the largest part of the area covered by the SAR scenes lies within the Yamalo-Nenets Autonomous Okrug. Smaller parts of the extents lie within the Nenets Autonomous Okrug and the Komi Republic. All three are federal territories of the Russian Federation. Nearly all of the study area is underlain by continuous permafrost, with only very small portions of discontinuous and sporadic permafrost, according to the maps by Brown et al. (1998). The vast majority of the studied lakes are located on the Yamal Peninsula. The Peninsula has been the subject of a variety of environmental studies. The primary long-term permafrost monitoring site at Vaskiny Dachi is located in its centre (Leibman et al. 2015; Dvornikov et al. 2016). Climate change and the associated increase in air temperatures is causing degradation of the permafrost layer on Yamal (Leibman et al. 2015). Past and present gas exploitation is leading to land-use and land-cover changes throughout the Peninsula (Kumpula et al. 2012). The Yamal Peninsula is the homeland of the largest remaining nomadic pastoralist group active in the Arctic, the Yamal Nenets. Direct human impact and climatic changes are largely affecting the grazing habitats of reindeer herds belonging to the Nenets (Forbes 1999; Forbes et al. 2009). The Yamal Peninsula shows an abundance of waterbodies, with a wide occurrence of thermokarst lakes (Kornienko 2017). One of the greatest densities of lakes in the Arctic occurs on Yamal (Paltan, Dash, and Edwards 2015). Within this study, more than 50,000 lake units were considered.

### 2.2. Sentinel-1 SAR data

The radar satellites Sentinel-1A (launched in April 2014) and Sentinel-1B (launched in April 2016) are part of the EU’s Copernicus Program and carry an identical SAR instrument, the C-SAR. The two satellites share the same polar, sun-synchronous orbit, separated by 180${\;}^{\circ }$ in the orbital plane (ESA 2013). C-SAR can be operated in different beam modes with varying spatial resolutions and swath widths. It can also be run in different polarization modes. The largest swath width is used in Extra Wide (EW) swath mode, with approximately 400 km, where a resolution of 40 m is achieved (Snoeij et al. 2011). The incidence angle range is 20${\;}^{\circ }$ to 45${\;}^{\circ }$ in EW mode. In EW and also in Interferometric Wide (IW) swath mode, the Terrain Observation with Progressive Scans SAR (TOPSAR) technique is used, which is a ScanSAR technique, where the beam is steered in range and azimuth direction, leading to a better quality of the SAR images.

The default operating mode is IW (spatial resolution of about 10 m) with VV and vertical-horizontal (VH) polarization over land, which satisfies most service requirements (ESA 2013). EW mode is primarily used for wide area coastal monitoring including ship traffic, oil spill, and sea-ice monitoring (ESA 2013). Sentinel-1 EW scenes often extend further south of the Arctic Ocean. In EW mode, scenes are mostly acquired in HH and horizontal-vertical (HV) dual-polarization mode or in HH single-polarization mode over arctic landmasses (Schmuck, Roeder, and Potin 2014). This includes the Yamal peninsula.

**Figure 1.**
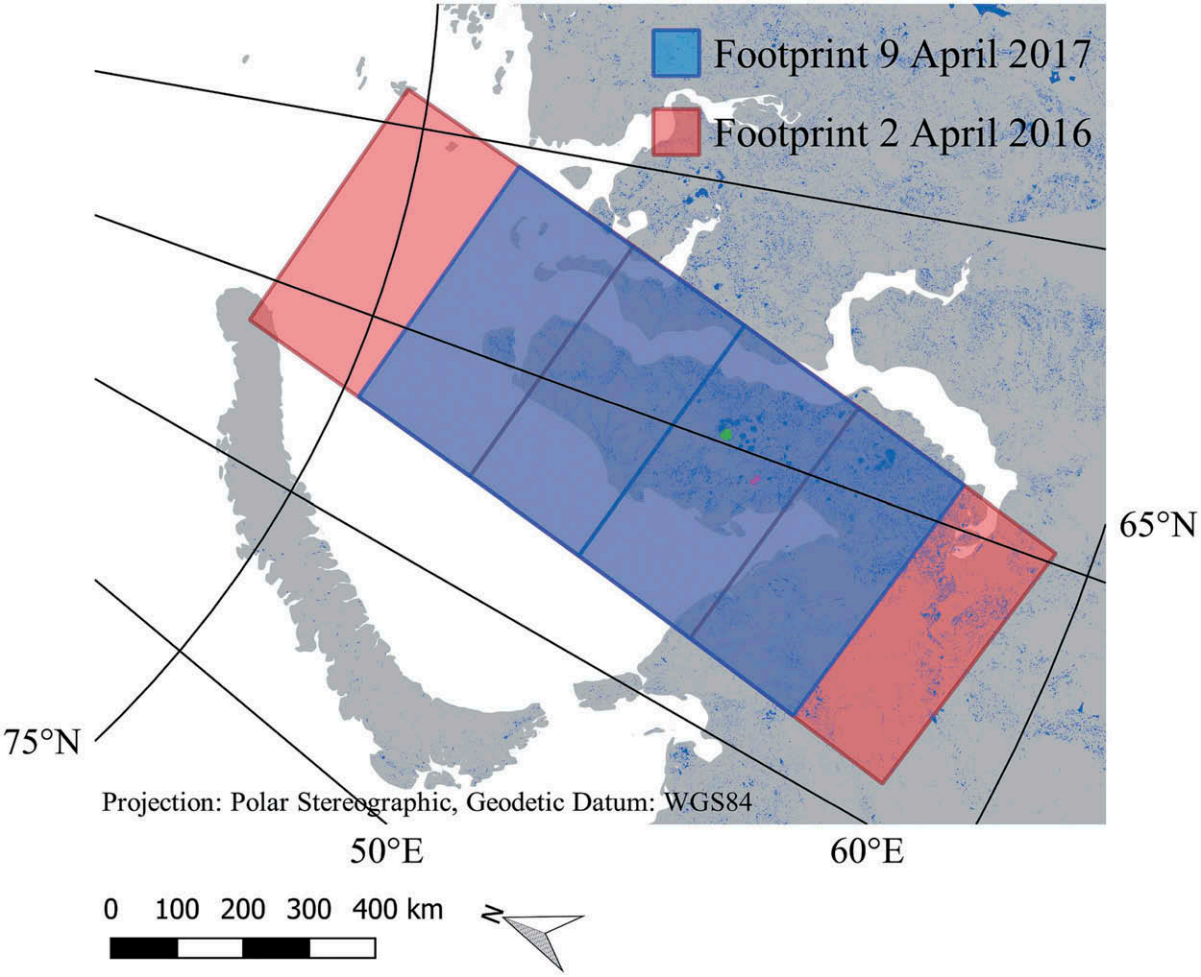
Coverage of Sentinel-1 scenes used for classification; scenes acquired on 2 April 2016 in red; scenes acquired on 9 April 2017 in blue; data source: European Space Agency (ESA). Lake Neyto in green and lake Yambuto in magenta (data source: National Geomatics Center of China).

For 2016, three adjacent scenes acquired on 2 April, covering nearly the whole Yamal Peninsula were selected. For 2017, two adjacent scenes of the same orbit acquired on 9 April were used. Figure 1 shows the coverage of the Sentinel-1 scenes used for classification. Only lakes in the overlapping area from both years were considered in the analysis.

In addition to the scenes used for the classification, several EW HH-polarized scenes from February to June 2016 and 2017 were used for a visual interpretation of specific patterns that cause differences between the results obtained with the different methods.

### 2.3. Sentinel-2 optical data

Sentinel-2 is the second pair of earth observation satellites of the EU’s Copernicus program. Sentinel-2A was launched into orbit in June 2015 and carries a multispectral imager, simply called MultiSpectral Instrument (MSI), which acquires images in 12 spectral bands (Drusch et al. 2012). The spatial resolution depends on the spectral bands and is 10 m for the optical red-, green- and blue channels. Sentinel-2B, equipped with an identical instrument, was launched in March 2017.

Sentinel-2 data were used to assess similarities of patterns between SAR and optical acquisitions. Sentinel-2 ‘true-colour’ composites (using the bands 4, 3, and 2) were obtained for that purpose. At high latitudes, a high percentage of cloud cover in scenes is very common, leaving only a few useful acquisitions throughout the year.

In optical images from summer, when the lakes are free of ice, the outlines and also the shallow shelves of many arctic lakes are often clearly visible. Since the freezing depths vary from year to year, the images can only give hints where ground-fast ice might have formed and where most likely not. The shallow shelf regions, where ground-fast ice usually forms in the winter, are often seen as brighter rings around the lake margins in optical summer images (Jones et al. 2017). For a visual comparison to the classification results and identifying shallow shelf regions for a quantitative comparison, ice-free scenes from 7 July 2016 were used.

Sentinel-2 optical images acquired when the lakes were partly free of snow in late winter showed interesting features on the lake surfaces, similar to those found in the SAR imagery. For a comparison to patterns observed in the SAR imagery, scenes from 21 May 2016 and 8 June 2017 were selected.

### 2.4. Lake mask

A lake mask originally obtained from the 30 m Global Land Cover 30 (GLC30) dataset of the National Geomatics Center of China (NGCC) was used to extract the lakes from the SAR imagery. Its full derivation is described in Bartsch et al. (2017). The GLC30 is the first global land cover dataset at a spatial resolution of 30 m, containing a comparable amount of classes. The dataset was obtained by NGCC through an advanced classification algorithm on the Landsat Thematic Mapper (TM), Landsat Enhanced Thematic Mapper+ (ETM+) and Chinese Environmental Disaster Alleviation Satellite (HJ-1) multispectral images. The GLC30 dataset is a static land cover map for the reference year 2010. To obtain a lake mask from it, reclassification was performed to extract only the waterbody class and this extracted class was vectorized. Large river- and bay-polygons were removed manually from the resulting vector dataset. This lake mask was used to mask the lakes in both years, 2016 and 2017.

## 3. Methods

### 3.1. Pre-processing of sentinel-1 SAR data

Wet snow or liquid water on top of the lake ice layer results in backscatter similar to ground-fast lake ice. Only frozen days need to be therefore considered. Daily maximum air temperatures recorded at the weather station Marre-sale (located on the west coast in the south of the Yamal Peninsula) were used as an indicator if melting processes took place during acquisitions. On 2 April 2016, the maximum air temperature was −5.4 ${\;}^{\circ }$C, the minimum temperature was −11.1 ${\;}^{\circ }$C. On 9 April 2017, no maximum temperature record was available, but the minimum temperature on that day was −25.9 ${\;}^{\circ }$C. On 8 April and 10 April 2017, the maximum temperatures were −13.4 ${\;}^{\circ }$C and −6.4 ${\;}^{\circ }$C, respectively (Menne et al. 2012b,2012a). Visual inspection was performed in addition for signs of backscatter that might be affected by melting snow.

The Sentinel Application Platform (SNAP) toolbox of the European Space Agency (ESA) was used for pre-processing of all SAR images. First, thermal noise and border noise were removed. Thermal noise can be seen as radiometric disturbances at the edges of the subswaths used by the TOPSAR system. Those disturbances were found to be significantly more striking for cross-polarized data (Park, Korosov, and Babiker 2017), but thermal noise removal is also considered to improve the quality of co-polarized data. Border noise can be easily identified visually on Sentinel-1 Ground Range Detected (GRD) scenes. Dark pixels are found at the borders of the images, that provide no useful information, so an automatic removal of border noise in SNAP was performed. However, there were still some erroneous pixel values remaining. Thus, all lakes in the affected border zone were removed manually from the lake mask and excluded from further analyses. Afterwards, the scenes were radiometrically calibrated to $\sigma^{0}$ at linear scale, terrain correction was performed using the Global Earth Topography And Sea Surface Elevation 30 (GETASSE30) Digital Elevation Model (DEM) and the projected local incidence angle was extracted. Backscattering coefficients were converted to units of decibels (dB). The output of the pre-processing were images containing $\sigma^{0}$ in dB and local projected incidence angle θ in ${\;}^{\circ }$ for each scene.

### 3.2. Classification

Three approaches were compared: threshold classification and two methods which take topology into account. It is typical for thermokarst lakes to only form ground-fast ice in the shelf regions, or over their entire extent, if they are very shallow. This has been exemplified using bathymetric data in various regions, e.g. the North Slope by Grunblatt and Atwood (2014), as well as central Yamal (Bartsch et al. 2017).

#### 3.2.1. Threshold classification

The method for the incidence angle dependent threshold classification was outlined and evaluated with bathymetric data in Bartsch et al. (2017). From the Sentinel-1 EW HH-polarized scenes, samples of $\sigma^{0}$ and local projected incidence angle θ were taken for the lakes for ground-fast and floating lake ice classes in order to determine the incidence angle dependence of the backscatter. Due to the relatively high difference in backscatter and the usual forming of ground-fast lake ice either along the shoreline or over the entire lake area, samples were selected based on visual inspection of the SAR scenes, which is a common procedure for these types of data (e.g. Hirose et al. 2008). The selected samples represent the whole incidence angle range. Second-degree polynomial functions were fitted to the data points for both, floating and ground-fast ice classes. The mean of these two functions was used as a threshold-classification function.

**Figure 2.**
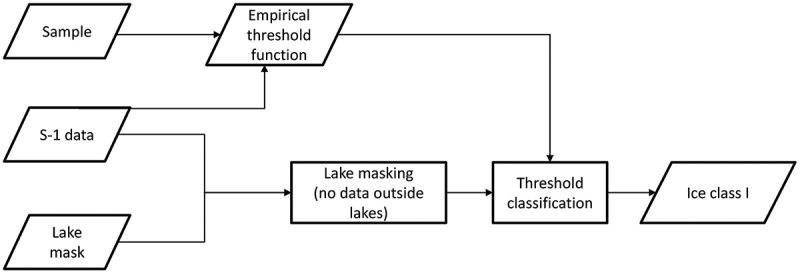
Flow chart of the threshold method algorithm applied to Sentinel-1 (S-1) data.

The images were masked with the GLC30 lake mask as a first step. For each scene, a pixel-based threshold classification using the obtained threshold function was performed: The threshold was set individually for each pixel using the $\sigma^{0}$-value of the threshold-function at the pixel’s incidence angle θ. If the pixel’s $\sigma^{0}$-value was below the threshold, it was classified as ground-fast ice, if it was above the threshold, it was classified as floating ice. Afterwards, a mosaic was produced by overlaying all classified scenes. Figure 2 shows a flow chart diagram of the threshold method algorithm.

#### 3.2.2. Flood-fill method

**Figure 3.**
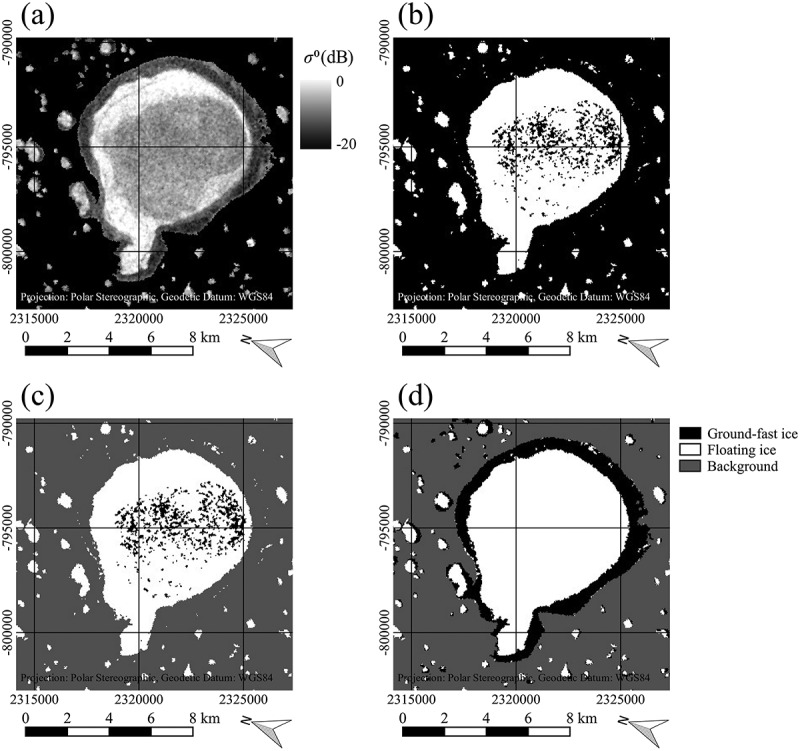
Illustration of the flood-fill method on a subset of a Sentinel-1 EW image, acquired on 2 April 2016, data source: European Space Agency (ESA); (a) Masked SAR image with background pixels in black, (b) Image after threshold classification, (c) Image after classification and flood-fill from the background region, (d) Final classification result. Contains modified Copernicus Sentinel data 2016, processed by ESA.

**Figure 4.**
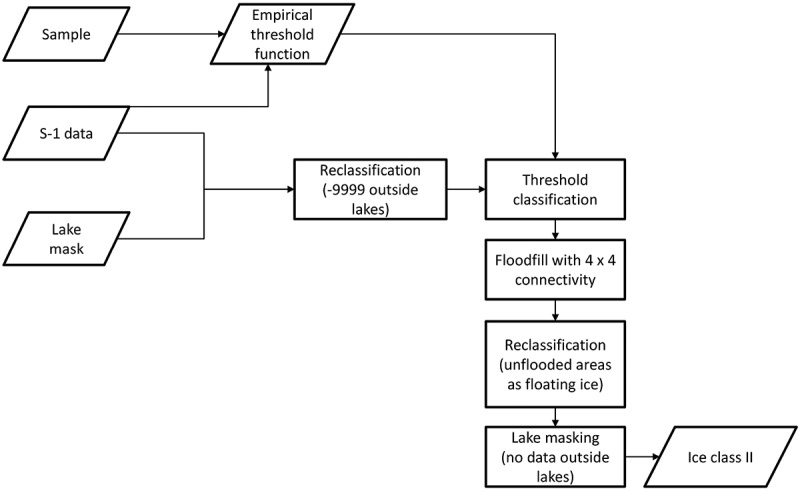
Flow chart of the flood-fill method algorithm applied to Sentinel-1 (S-1) data.

In case of the Flood-fill method, the image was masked with the GLC30 lake mask before the classification to set the background no-data pixels. The principle is illustrated using a small subset containing one relatively large lake (Figure 3). The background no-data pixels from the masked scene were set to a relatively low value first (Figure 3(a)). Then, the image was classified just as in the threshold method with the obtained threshold function. Afterwards, because of the low value of the background pixels before, the background pixels and the ground-fast pixels belonged to the same class (Figure 3(b)). In the following, a flood-fill with a 4-pixel-connectivity (Heckbert 1990) was used, which filled all connected background and ground-fast pixels with the same value starting from the first background pixel in the upper left of the image (Figure 3(c)). Unconnected pixels within the lakes remained unchanged. Finally, the initial background values could be used to distinguish between background and the ground-fast ice class (Figure 3(d)). Figure 4 shows a flow chart diagram of the flood-fill method algorithm.

#### 3.2.3. Watershed method

The second new method relies on a marker-based watershed segmentation. Segmentation is usually used to define image objects, groups of pixels with similar properties, that are classified in a separate step by using a selected algorithm (e.g. Wang, Sousa, and Gong 2004). Here in this approach, segmentation and classification are virtually performed in one step, as only two classes have to be distinguished. The method is somewhat related to the flood-fill method, as its goal is also the discrimination between shallow shelf areas where ground-fast ice is present and areas of floating lake ice outside the shelves.

The initial method of the watersheds transformation for image segmentation was introduced by Digabel and Lantuéjoul (1978). Any grey-scale image can be considered as a topographic surface, where the intensity of a pixel represents its elevation. A watershed is a line that determines where a drop of water will fall into a particular region considering the image as a topographic relief. The basic principle can be described by water that is used to flood this topographic surface (Meyer and Maragos 1999). If the relief is flooded from its minima and water from different regions is prevented from merging, the image will be partitioned in watershed lines and catchment basins. The watershed lines represent the boundary between water from different regions and the catchment basins are the regions enclosed by the watershed lines. However, defining the minima is not always straightforward and therefore a major enhancement of the method is the marker-based watershed segmentation. Here, the surface is not flooded from its minima, but from predefined regions within the image, the markers. The numbers of identified catchment basins are equal to the number of used markers. The marker-based watershed segmentation has been found to be a robust and flexible method for segmentation of objects with closed contours, where the boundaries are expressed as ridges (Parvati, Rao, and Mariya Das 2008). It is also expected to be a useful tool to identify the boundary between ground-fast lake ice in the shelf areas and floating lake ice from SAR images.

**Figure 5.**
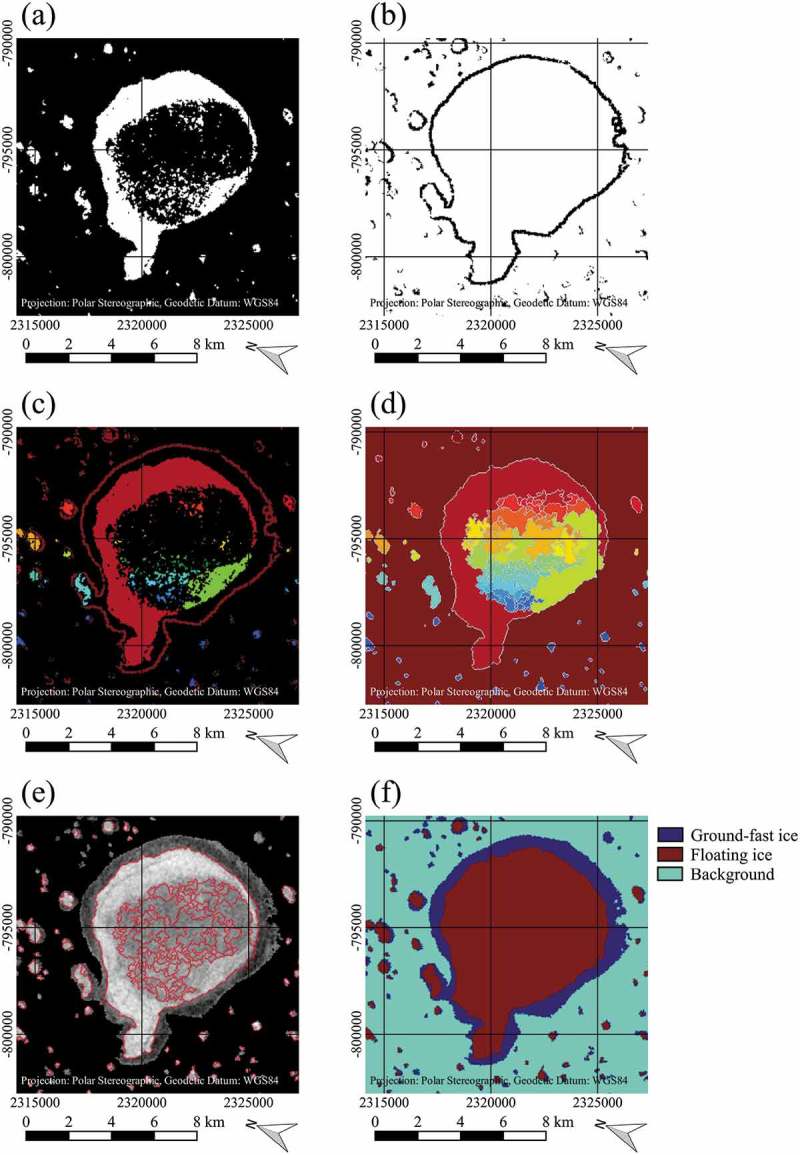
Illustration of the watershed method on a subset of a Sentinel-1 EW image, acquired on 2 April 2016, data source: European Space Agency (ESA); (a) Sure floating lake ice pixels in white, (b) Sure ground-fast lake ice pixels in black, (c) Markers for watershed segmentation (marker for the ground-fast ice class in darkest red), (d) Watershed lines (white) and catchment basins (coloured, the darkest red represents the catchment basin for background and ground-fast ice pixels) after segmentation, (e) Watershed lines (red) on top of initial SAR image, (f) Final classification result. Contains modified Copernicus Sentinel data 2016, processed by ESA.

The implementation of the algorithm involves a number of steps and is also described for the same small example area as for the flood-fill method (Figure 5). In contrast to the previously described methods, $\sigma^{0}$ of each pre-processed Sentinel-1 EW scene was normalized to a common reference incidence angle of 30${\;}^{\circ }$ using:

(1) $$\sigma^{0}(30)=\sigma^{0}(\theta )-(\sigma_{thresh}^{0}(\theta )-\sigma_{thresh}^{0}(30))$$

where $\sigma_{thresh}^{0}(\theta )$ is the derived threshold function and $\sigma^{0}(\theta )$ is the backscatter coefficient of a pixel at its corresponding projected local incidence angle. It considers backscatter coefficients in dB. Backscatter varies by approximately 5.5 dB between 20${\;}^{\circ }$ and 40${\;}^{\circ }$ incidence angle for both, floating and ground-fast ice. For this type of normalization, variations of the differences between the two ice types with incidence angle and hence variations of the differences between the threshold function and the functions fitted to the two sample classes with incidence angle have to be considerably small. The difference between the threshold function and the functions fitted to the sample classes at 20${\;}^{\circ }$ (lower end of incidence angle range) is 4.4 dB, at 40${\;}^{\circ }$ (upper end of incidence angle range) it is 4.3 dB.

**Figure 6.**
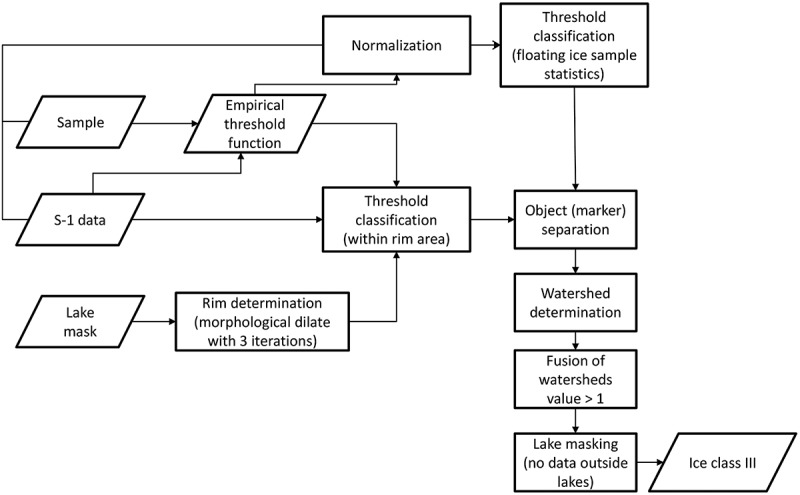
Flow chart of the watershed method algorithm applied to Sentinel-1 (S-1) data.

The lakes were masked with the lake mask described above and background values were set to a relatively low value (same calculation as in the flood-fill method, see Figure 3(a)). All pixels above a statistically derived threshold ($th_{ice}$) based on analysis of the samples which were used for the determination of the threshold function were considered to surely belong to the floating ice class (white pixels in Figure 5(a)). The mean of the normalized ground-fast ice samples minus three times the standard deviation has been used. This value also lies above 99.9% of all normalized ground-fast ice samples. Morphological dilate with a quadratic kernel of 3 × 3 pixels and three iterations was applied to create a buffer zone from the lake outlines. All pixels within this buffer zone below a further threshold $th_{water}$, which corresponds to the backscatter at 30${\;}^{\circ }$ using the initial threshold function (Section 3.2.1.) were considered to belong to the ground-fast ice class (black pixels in Figure 5(b)). All connected sure floating ice pixels were defined as markers and assigned an index starting with 2 ascending. All sure ground-fast ice pixels were used as one marker and assigned the index 1. All other pixels were defined as unknown. These pixels were the target pixels to be identified by the watershed algorithm. Figure 5(c) shows the unknown pixels in black and the markers in different colours depending on their index on a scale from red to blue. The sure ground-fast marker with index 1 is shown in the darkest red. In the following, the watershed algorithm was applied to the image with the defined markers. Figure 5(d) shows the catchment basins identified from each marker in different colours and the watershed lines in white. Figure 5(e) shows the watershed lines in red on top of the original image. Initial background pixels and ground-fast ice pixels in the shelf area are covered by one large catchment basin (the darkest red in Figure 5(d)) obtained from the marker with index 1. All other catchment basins were then classified as floating lake ice. Knowing the initial background pixels then again allowed to distinguish them from the ground-fast ice class (Figure 5(f)). Figure 6 shows a flow chart diagram of the watershed method algorithm.

### 3.3. Evaluation

In order to analyse differences between the three classification approaches, total fractions and distributions of identified ground-fast lake ice area for different lake size classes were computed for each approach and compared to each other and between 2016 and 2017.

Lakes with extensive differences between the methods have been identified and visually compared to optical data from Sentinel-2. Different spatial patterns that cause partially large differences between the methods were identified.

In order to determine classification accuracy, 20 lakes were chosen, where shallow shelf areas (bright colour along the outlines) and wide deeper central parts (darker blue or green colour) could clearly be distinguished in the Sentinel-2 summer imagery. One hundred metre distance buffer zones were calculated from the outlines inward for each of the 20 lakes to represent the shelf areas, where the potential occurrence of ground-fast ice is assumed. Centroids were calculated for each of the 20 lakes, and circles with 1 km diameter around these centroids were computed to represent the deeper areas, where only the occurrence of floating ice is expected. The percentages of ground-fast and floating ice detected by the three methods were calculated for each of the shelf- and central zones.

Results have been also compared to a similar dataset derived from ENVISAT ASAR Wide Swath (WS) mode data (Bartsch et al. 2017). It was calibrated and validated with bathymetric data from central Yamal. Its nominal resolution is 75 m and represents April 2008. The same lake mask was applied for retrieval.

## 4. Results

When performing a threshold-based classification similar to that used in Bartsch et al. (2017), it was found that clusters of pixels, predominantly located in central parts of the largest lakes, might be falsely classified as ground-fast lake ice. Correctly identified ground-fast ice pixels are considered to be located in the shelf regions and connected to all other ground-fast ice pixels along the lake margin. However, the boundary between ground-fast and floating lake ice can be made out rather easily in the original backscatter images and appears to be classified correctly with the threshold method in many cases.

In 2016, the total fractions of ground-fast ice detected from the 20 selected lakes in the shelf zones are 92.6% for the threshold, 92.6% for the flood-fill and 96.7% for the watershed method; the fractions of ground-fast ice in the centre zones are 11.3% for the threshold, 4.7% for the flood-fill and 0.0% for the watershed method. In 2017, the total fractions of ground-fast ice detected in the shelf zones are 96.2% for the threshold, 96.1% for the flood-fill and 97.3% for the watershed method; the fractions of ground-fast ice in the centre zones are 7.6% for the threshold, 0.0% for the flood-fill and 0.0% for the watershed method.

**Figure 7.**
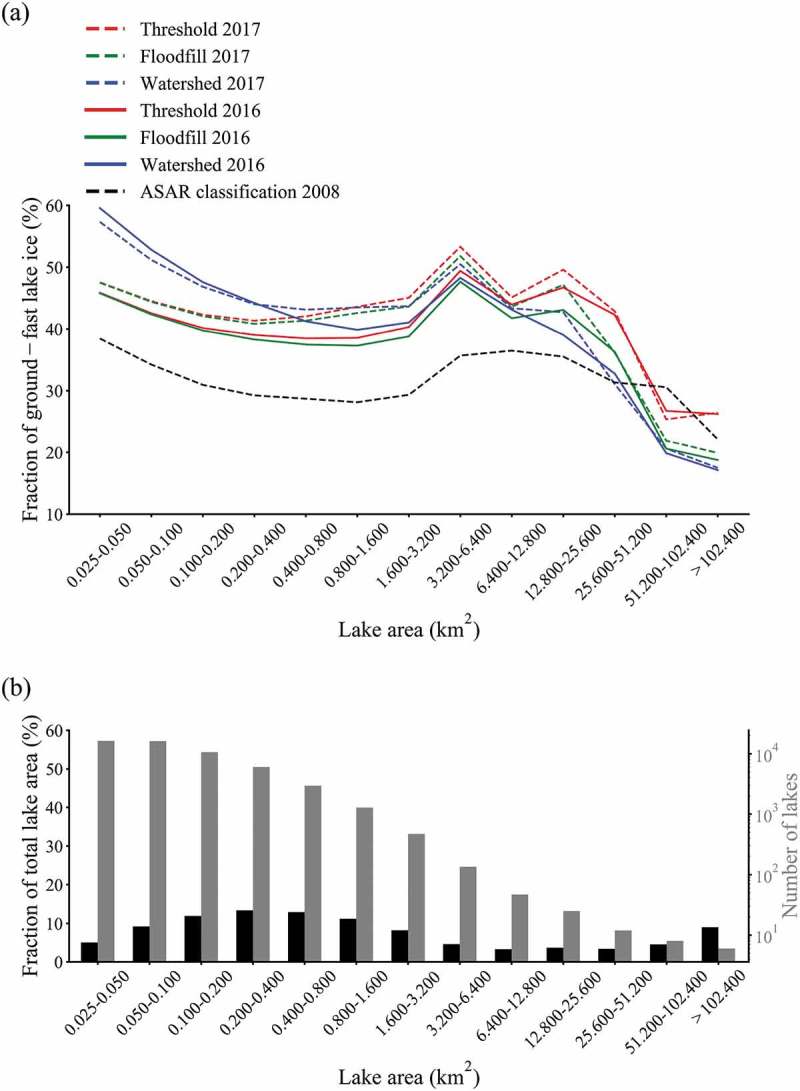
Lake ice properties for lake size classes. (a) Comparison of detected ground-fast ice fractions from Sentinel-1 for lake area classes by different classification methods of 2 April 2016 (continuous lines) and 9 April 2017 (dashed lines), averaged for lake size categories. Black dashed line represents the ENVISAT ASAR WS threshold classification of Bartsch et al. (2017) (b) Additional information for lake size classes. The bars indicate the fraction of the total lake area (black) and the number of lakes (grey) for the respective lake size class (data source: National Geomatics Center of China).

Fractions of ground-fast lake ice differ for the size classes (Figure 7(a)). Between 0.025 km${\;}^{2}$ and 12.800 km${\;}^{2}$, fractions identified by the threshold and flood-fill method are essentially similar, broadening slightly with increasing lake size. This implies that the amount of ground-fast pixels identified in the shelf areas by the flood-fill method and overall identified ground-fast ice pixels by the threshold-method might be similar.

The watershed method detected significantly larger amounts of ground-fast ice pixels for lakes smaller than 1.600 km${\;}^{2}$. The largest difference is observed for the smallest lake size class (more than 15.0%) and narrows with increasing lake size. Clusters of pixels presumably wrongly classified as ground-fast were visually only identified for the largest lakes. The coarser resolution classification from ASAR WS provides lower values but a similar shape of distributions for lakes smaller than 25.600 km${\;}^{2}$.

The fractions detected above 12.800 km${\;}^{2}$ are similar between flood-fill and watershed method, with larger deviations from the threshold method. It is assumed that the mentioned effects of pixels falsely classified as ground-fast in central parts of the larger lakes are widely reduced by the flood-fill and watershed methods and that this effect is responsible for the deviation. The difference to the threshold approach is especially large for the largest two size classes. Although only 14 lakes fall within these classes, they make up more than 10.0% of the total lake area studied (Figure 7(b)).

As can be seen in Figure 7(a), in general, and specifically for the lakes in the medium size range, the detected average fraction ground-fast lake ice is slightly higher in 2017 than in 2016. For the three largest size classes, detected fractions are much more similar to each other than in the medium size range.

**Figure 8.**
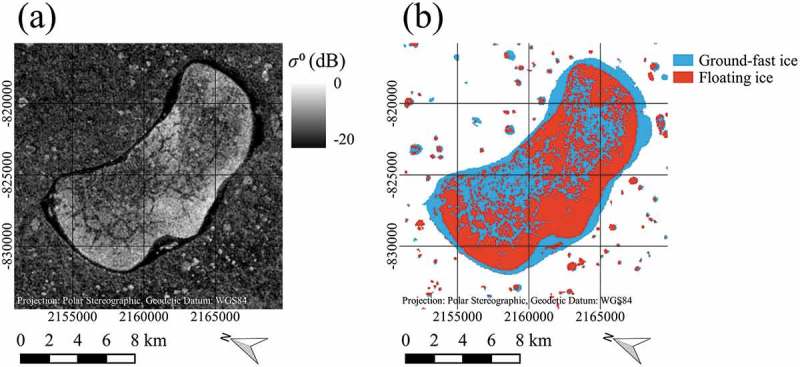
Comparison between Sentinel-1 SAR image and threshold classification result for lake Yambuto, acquired on 9 April 2017, data source: European Space Agency (ESA); (a) Sentinel-1 SAR image (b) Result of threshold classification. Contains modified Copernicus Sentinel data 2017, processed by ESA.

In a visual inspection of the classification results and the original SAR scenes, 3 major types of features that cause significant differences between the results of the different classification methods for large lakes could be identified. Some of the lakes show wide areas in the assumed floating ice region, where backscatter is significantly lower than in the surroundings and in other lakes. These wide patches are thought to cause large classification errors, especially with the threshold method. An example of this phenomenon can be seen in Figure 3, which was already used to illustrate the principle of the flood-fill method. Here, the very central part of the lake exhibits significantly lower backscatter values than the surrounding part. Another example can be seen in Figure 8, which shows the original SAR scene of lake Yambuto and the associated threshold classification result of 2016. Wide patches of low backscatter, which are assumed to be part of the floating ice regime are evident in Figure 8(a). In Figure 8(b), it can be seen that these patches presumably caused large errors in the result of the threshold classification. Also, the second type of feature can be identified, which are relatively straight, narrow lines of low backscatter. These lines appear like cracks in the ice, causing presumably errors in the threshold classification of smaller spatial scale.

**Figure 9.**
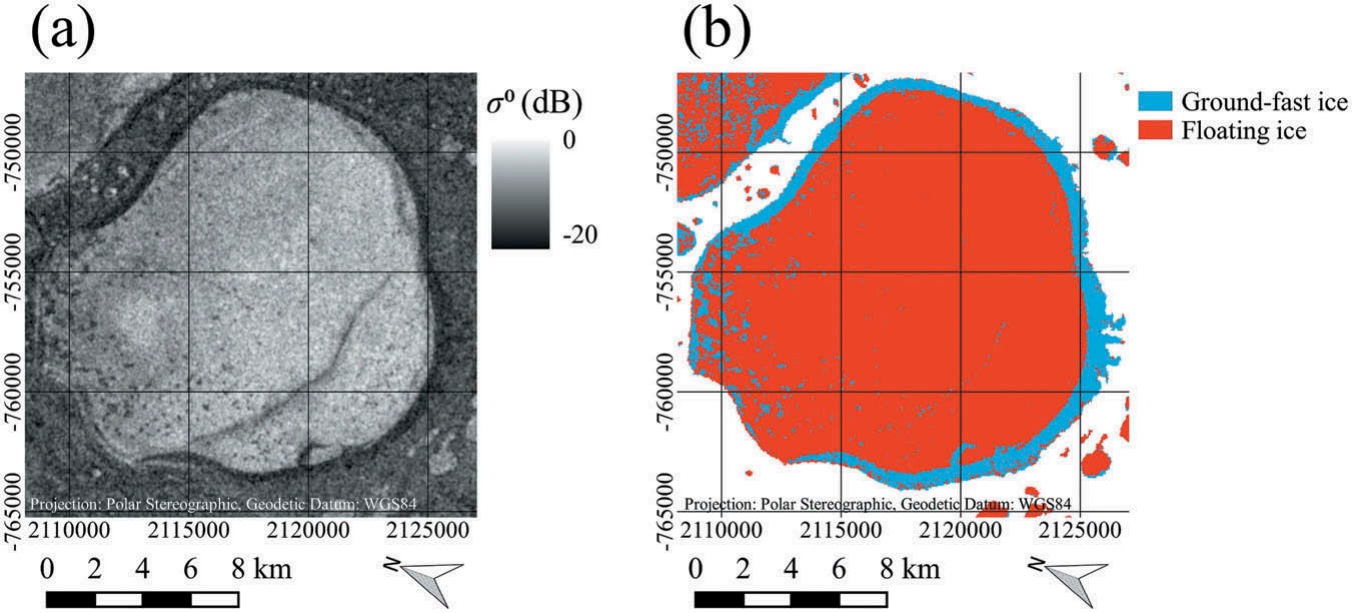
Comparison between Sentinel-1 SAR image and threshold classification result for lake Neyto, acquired on 2 April 2016, data source: European Space Agency (ESA); (a) Sentinel-1 SAR image (b) Result of threshold classification. Contains modified Copernicus Sentinel data 2016, processed by ESA.

Another line of low backscatter, that appears to be a crack within the ice, can be seen in Figure 9, which shows the original SAR scene of lake Neyto and the associated threshold classification result of 2017. Here, also a third type of identified features can be seen. A pattern of dot-like regions of low backscatter can be identified in Figure 9(a), which are clearly reflected by the threshold classification result (Figure 9(b)).

**Figure 10.**
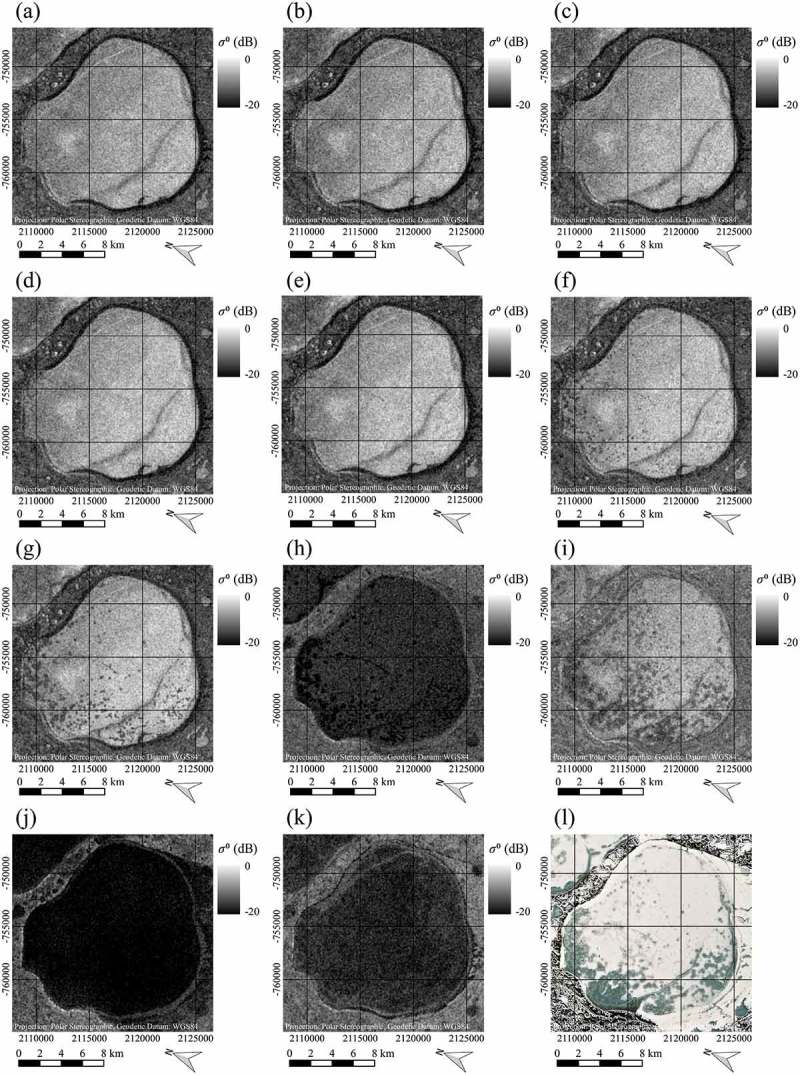
Time series of Sentinel-1 SAR images indicating occurrence and changes of dot-like patterns of low backscatter and comparison to partly snow-free Sentinel-2 ‘true-colour’ composite of lake Neyto in 2016, data source: European Space Agency (ESA). SAR images acquired on: (a) 2 February 2016, (b) 14 February 2016, (c) 26 February 2016, (d) 9 March 2016, (e) 21 March 2016, (f) 2 April 2016, (g) 14 April 2016, (h) 26 April 2016, (i) 8 May 2016, (j) 20 May 2016, (k) June 1 2016. Sentinel-2 ‘true-colour’ composite (l) acquired on 21 May 2016. Contains modified Copernicus Sentinel data 2016, processed by ESA.

**Figure 11.**
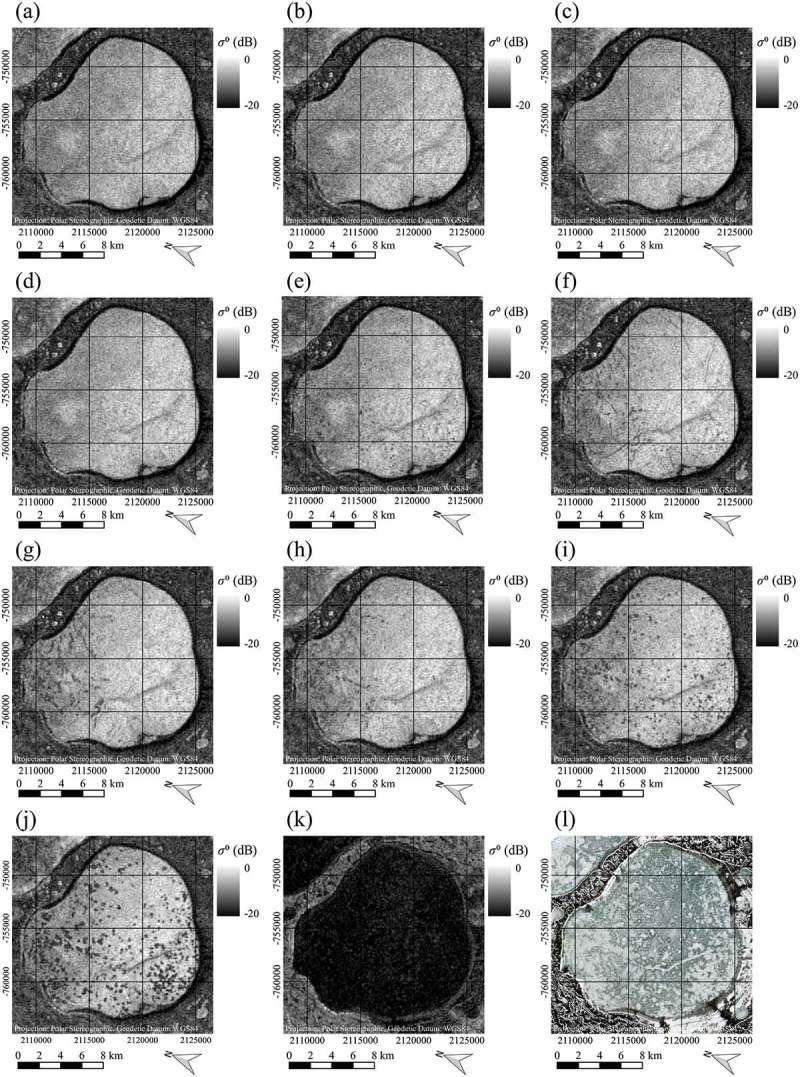
Time series of Sentinel-1 SAR images indicating occurrence and changes of dot-like patterns of low backscatter and comparison to largely snow-free Sentinel-2 ‘true-colour’ composite of lake Neyto in 2017, data source: European Space Agency (ESA). SAR images acquired on: (a) 8 February 2017, (b) 20 February 2017, (c) 4 March 2017, (d) 16 March 2017, (e) 28 March 2017, (f) 9 April 2017, (g) 21 April 2017, (h) 3 May 2017, (i) 15 May 2017, (j) 27 May 2017, (k) 8 June 2017. Sentinel-2 ‘true-colour’ composite (l) acquired on 8 June 2017. Contains modified Copernicus Sentinel data 2017, processed by ESA.

These ‘dots’ are especially interesting, as they clearly stand out against the surrounding much brighter pixels. Further visual investigations of SAR time series for lake Neyto showed that these patterns change throughout the winter season, becoming more apparent and larger with time, and also differ strongly when compared between the two years (Figures 10 and 11(a-k) respectively). In Figures 10(l) and 11(l), Sentinel-2 images acquired approximately when the lakes became free of snow cover can be seen for comparison. While for the year 2016 in Figure 10(l) the lake appears to be covered largely with snow and only smaller darker patches of the lake appear snow-free, for the year 2017 in Figure 11(l) the lake appears to be nearly entirely free of snow, showing some brighter patches in the ice. Interestingly, the snow-free patches in 2016 but also the brighter patches in 2017 resemble the patterns that can be seen in the earlier SAR acquisitions. In Figure 10(h,j,k), and in Figure 11(k) very low backscatter coming from the lake can be observed. This is very likely due to wet snow or liquid water on top of the lake ice and thus a comparison with the optical images is not reasonable. Because of high dielectric losses of water, C-band backscatter of snow decreases with increasing liquid water content (Nagler et al. 2016). At incidence angles above 10${\;}^{\circ }$, C-band backscatter of wet snow is significantly lower than for dry snow or snow-free areas (Nagler and Rott 2000).

## 5. Discussion

The similarity between the threshold and flood-fill method may not be surprising for the smallest lake size classes, as pixels of low backscatter are thought to be connected to each other anyway, but for the remaining lakes smaller than 12.800 km${\;}^{2}$ it is expected that ground-fast lake ice was largely identified correctly by both of those methods, since the similarity suggests that both methods largely succeeded in discriminating the shallow shelf regions from the deeper central lake parts.

The watershed method is thought to classify smaller areas of floating lake ice in central parts of small lakes incorrectly as ground-fast lake ice. This might be caused by the small number of central lake pixels that would belong to the floating ice class. If all of the few central pixels show comparably low backscatter values below −10.0 dB, the whole lake is classified as ground-fast.

For the larger classes (12.800 km${\;}^{2}$-25.600 km${\;}^{2}$), it is not quite clear which method should be preferred. Defining proper lake size limits for which methods are considered to work better might be beneficial for future classifications, but this has not been assessed yet.

**Figure 12.**
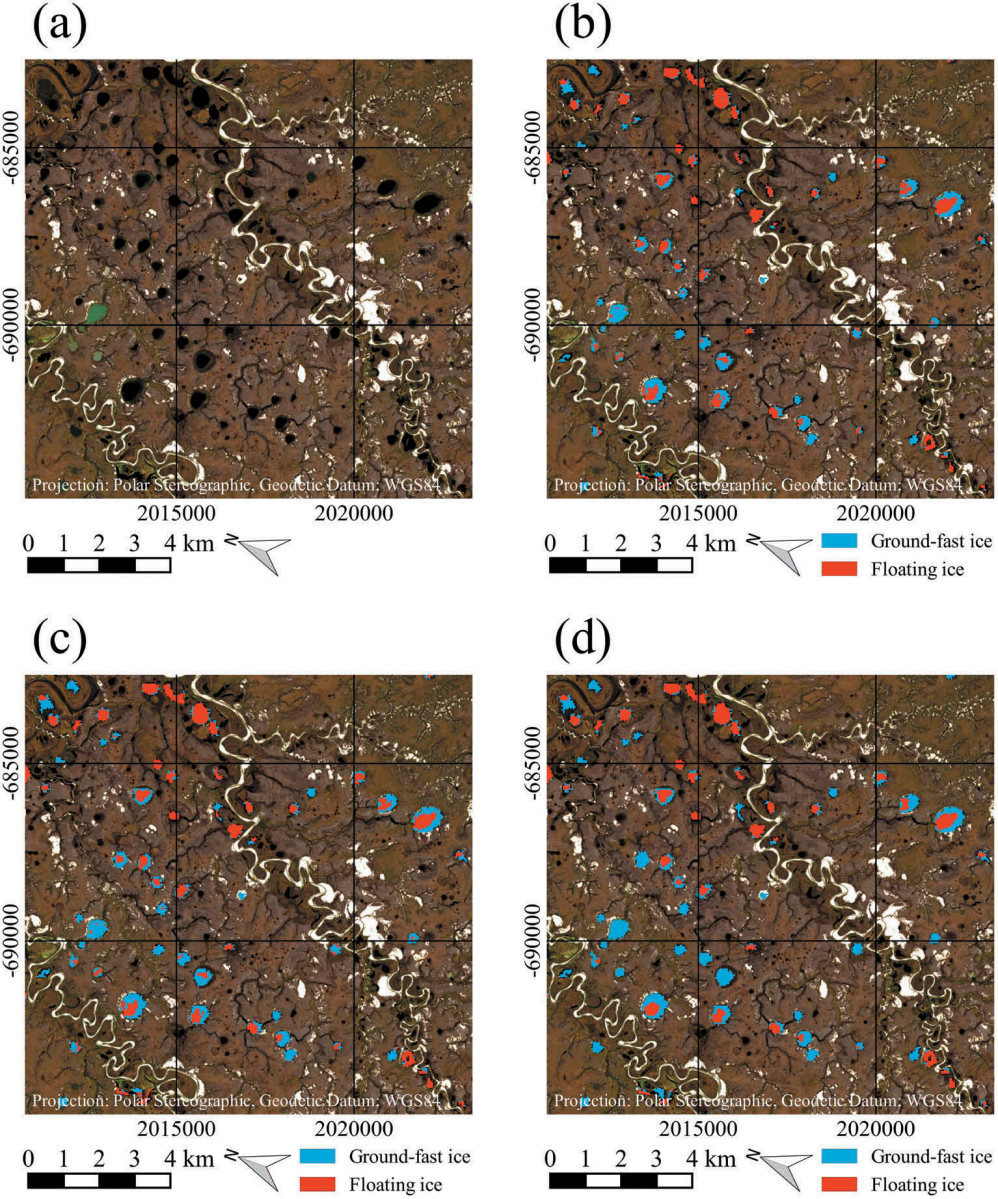
Example of the different classification results from 2016 for comparably small lakes on top of Sentinel-2 image, acquired on 7 July 2016, data source: European Space Agency (ESA); (a) Sentinel-2 ‘true-colour’ composite, (b) Result of threshold classification, (c) Result of flood-fill classification, (d) Result of watershed classification. Contains modified Copernicus Sentinel data 2016, processed by ESA.

In the following, two visual examples shall illustrate some points discussed above. Figure 12 shows the classification results of the three methods for comparably small lakes on top of a Sentinel-2 ‘true-colour’ composite. Except for very few pixels, the results of the threshold- and flood-fill methods are equal (Figure 12(b,c)). Nearly all pixels identified as ground-fast by the threshold method are connected to the lake outlines. Near the centre of Figure 12(d) and next to the river course, lakes can be seen that were entirely classified as ground-fast with the watershed method, in contrast to the flood-fill and threshold results, where small areas of floating lake ice are identified. It is assumed that the watershed method produces too high fractions of the ground-fast area and is not well applicable to smaller lakes.

**Figure 13.**
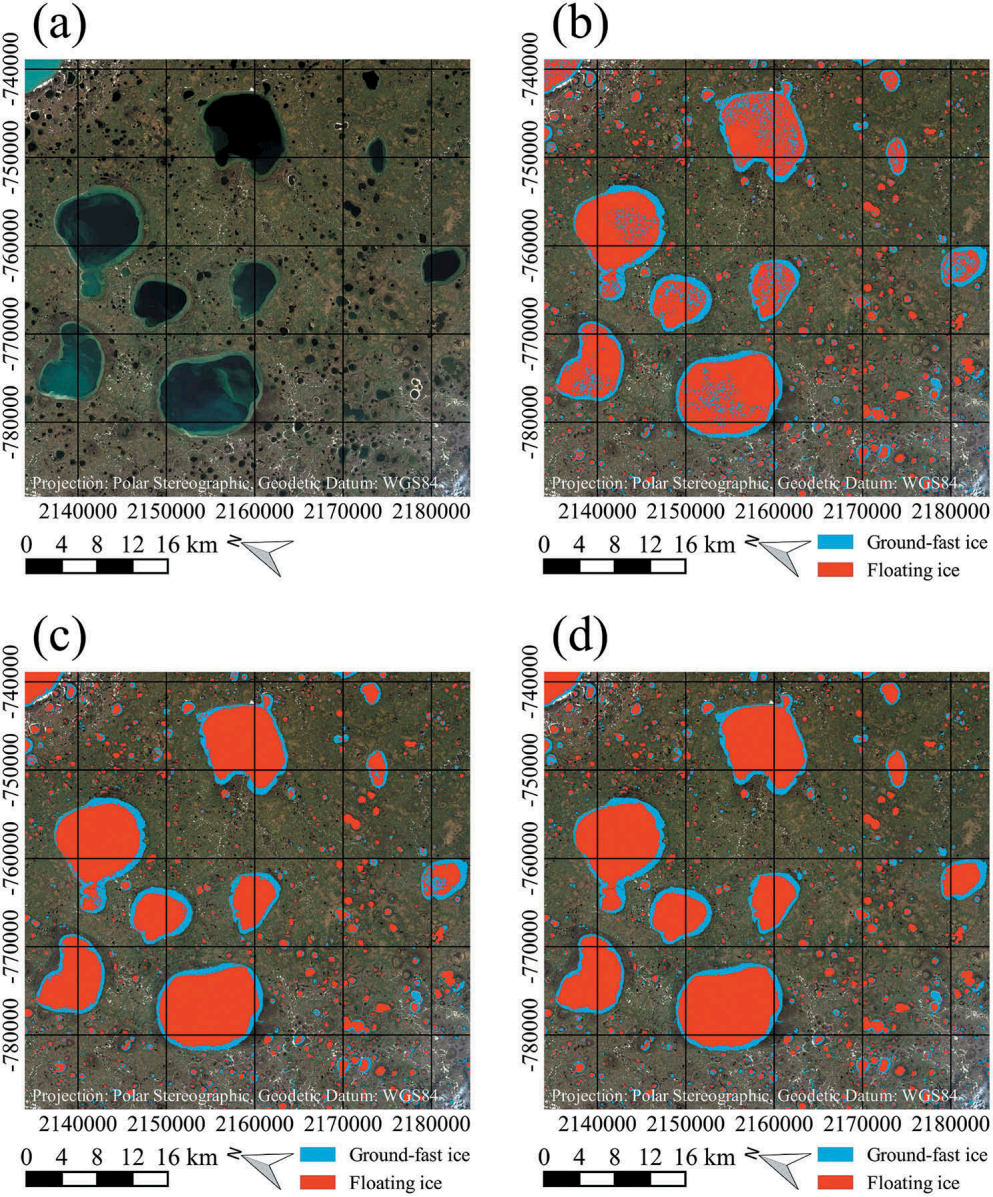
Example of the different classification results from 2016 for comparably large lakes on top of Sentinel-2 image, acquired on 7 July 2016, data source: European Space Agency (ESA); (a) Sentinel-2 ‘true-colour’ composite, (b) Result of threshold classification, (c) Result of flood-fill classification, (d) Result of watershed classification. Contains modified Copernicus Sentinel data 2016, processed by ESA.

Figure 13 shows the classification results of the three methods for comparably large lakes on top of a Sentinel-2 ‘true-colour’ composite. Shallow shelf areas can be identified rather easily in Figure 13(a). With the threshold approach, partly large amounts of pixels are classified as ground-fast in central parts of the large lakes, that are considered to be wrongly identified (Figure 13(b)). Significant improvements are considered to be obtained with the flood-fill (Figure 13(c)) and watershed (Figure 13(d)) methods. For the lake in the middle right of the images, presumably falsely classified pixels can be seen with the flood-fill method, as those pixels were connected to the ground-fast pixels along the shelf after the threshold classification, as can be seen from Figure 13(b). The watershed method presumably delivers a better result for this lake. A similar effect was also encountered for one lake belonging to the largest size class. This may lead to the assumption that the watershed method might provide the best result for the three largest size classes.

Winter 2017 was generally colder than average, including some periods of extreme cold. The minimum temperature recorded at the weather station Marre-sale was −39.0 ${\;}^{\circ }$C in the winter 2017 and −30.0 ${\;}^{\circ }$C in the winter 2016. Reindeer herders observed dead fish in case of shallow lakes, which they attributed to be the result of thicker ice, in many cases with lakes freezing all the way to the bottom. Local extinction of fish in otherwise normally productive lakes presents problems for Nenets tundra nomads, who depend on fish as their primary source of protein during the snow-free season across virtually all of Yamal Peninsula.

The used lake mask may introduce classification errors. It is static and from a different year than the Sentinel data. It has been shown by Trofaier et al. (2013) that the recession of inundation after snowmelt can last until August over the Yamal peninsula. The change in overall water fraction from July to August is in the order of 0.5% over central Yamal. It usually affects the rims of the lakes. The objects in the lake mask might be therefore too large in some cases. Affected lakes might be therefore classified as ground-fast ice along their rims due to the comparably low backscatter of the surrounding tundra (approximately −14.0 dB). This may lead to an overestimation of ground-fast lake ice. The case of disappearing or shrinking lakes over time, from year to year, is expected to have a similar effect. There might be, however, only very few cases within the six years. It is known that waterbodies change shape (often increase) due to human impacts (Yu et al. 2015) in the Yamal region. Those are usually shallow features (can be crossed by vehicles). An unaccounted increase of open water would result in an underestimation of ground-fast lake ice in such a case.

The lower ground-fast ice values of the ASAR WS product may result from the coarser spatial resolution. More mixed pixels are expected to occur along the lake rims. The higher ground-fast ice values for larger lakes point to the uncertainties due to different backscatter responses of ice in the lake centres.

The differences for the larger lakes underline the importance of analysing possible classification errors for the largest lakes. Three feature types that could be largely responsible for the classification errors with the threshold method were identified. For the line-type features, cracks might indeed be responsible for the observed low backscatter, which appears to be the case in a comparison to the optical data in Figures 10 and 11. For the wide area patterns, generally lower roughness in these areas could be responsible for the phenomenon, but these issues could not be assessed further within this study.

The comparison to optical data clearly indicates strong similarities between the dot-like patterns of low backscatter and the presumably snow-free areas of lake Neyto in 2016 seen in the optical acquisition in Figure 10(l), and also the bubble-like bright patches in Figure 11(l). One explanation for these patterns could be ebullition seeps of methane. Ebullition (bubbling) is often the dominant mode of methane release from Arctic lakes (Walter et al. 2006). Walter et al. (2006) describe a so-called hotspot-type of ebullition seeps, which are basically relatively open holes in ice due to extremely high bubble flux from discrete points in lake sediments. The convection of warm (0.0 ${\;}^{\circ }$C-2.0 ${\;}^{\circ }$C) lake water carried to the surface by bubbling leads to the holes in the ice (Anthony et al. 2010). Lindgren et al. (2016) describe that open-hole hotspots appear dark against the snow-covered lake in early-winter optical aerial images. Late-winter images have not been used to identify hotspots yet.

Walter et al. (2008) note, that while bubbles trapped in the ice tend to increase radar backscatter, hotspots likely influence the backscatter differently and this influence should be addressed in further studies. An open water surface or a water surface covered by a thin ice layer might cause mainly a specular reflection of the radar signal, leading to the observed patterns in the SAR imagery. However, the largest open-hole hotspots reported are about 300 m${\;}^{2}$ (Anthony et al. 2012), below the resolution of the Sentinel-1 EW SAR imagery. Greene et al. (2014) show that hotspot bubbling can maintain a large cavity where the gas accumulates under a thin layer of ice during the winter, but cavities in lake Neyto would still have to be very large to explain the observed patterns. Nevertheless, the white bubble-like bright patches in Figure 11(l) could indicate the presence of large cavities of gas. It is known from the area that methane emissions can cause a range of large-﻿scale features such as craters (Leibman et al. 2014; Kizyakov et al. 2017). They could be a possible reason for the observed pattern, but there is no direct observation, neither in the winter nor summer. Bogoyavlensky et al. (2016) describe a similar phenomenon for some lakes in central Yamal using high-resolution optical data (Worldview). Observations are similar to Sentinel 2 from 2016 in our study (Figure 10(l)). Snow free circular features are visible in the optical images but limited to 25–30 m diameter. Bogoyavlensky et al. (2016) interpret the phenomenon as a result of gas bubbles and suggest the use of such remote sensing images for exploration of gas resources from space.

Large circular patterns in lake ice, which can be identified with optical satellite data, have been also reported for Lake Baikal as well as Hovsgol (Kouraev et al. 2016). They develop during the second part of the winter, similarly to our observed patterns, but are much bigger, 2 km-8 km in diameter. In situ measurements showed that ice is thinner at these sites. Water temperature measurements suggested the development of eddies within the water, which affect the ice above and cause the circular shapes. Smaller temperature anomalies under ice have been reported by Forrest et al. (2013) and Kirillin et al. (2015). Eddy formation could therefore also be a potential explanation for the observed features. Especially the observed gradual development over the second part of the winter supports this hypothesis.

The occurrence of locally thin ice in central Yamal was also observed by Nenets. For example, reindeer herders reported in the summer 2017, that once they were crossing the large lake Yambuto (Figure 8) in mid-March, suddenly ice cracked under the first sledge in the middle of the lake. Ice thickness was just a few centimetres, and it was not visible as on top of the ice was thick snow cover. They were surprised as generally ice thickness at that time was more than one metre.

Much more research is needed to evaluate the origin of the mentioned patterns. The resemblance of the patterns in the SAR images with the presumably snow-free areas in Figure 10 (l), as well as with the white bubble-like patches in the presumably largely snow-free ice in Figure 11(l) has yet to be explained in detail. Visual interpretation on medium-resolution optical satellite imagery of late-winter is difficult and high cloud cover at these latitudes limit the number of Sentinel-2 acquisitions useful for the analysis of the phenomenon. Airborne measurements could be very useful for further studies. If the described patterns really represent ebullition seeps, Sentinel-1 data could be very useful for mapping large-scale methane release on Yamal in the future, especially when taking the 10-m resolution IW data into consideration.

## 6. Conclusions

Within this study, the role of lake size and the effects of local phenomena on discriminating ground-fast from floating ice on Arctic lakes on and near the Yamal Peninsula using Sentinel-1 SAR imagery of April 2016 and April 2017 was assessed. Arctic lakes are large emitters of carbon dioxide and methane, which is also released in the winter. Mapping the extent of floating and ground-fast ice accurately can be useful for emission studies. Moreover, it is important for understanding sublake permafrost dynamics and lake habitat changes. With the flood-fill and the watershed method, two new methods relying on image processing techniques were introduced. The two methods were designed to separate shallow shelf regions from central parts of the lakes in the SAR imagery because the threshold classification showed partly large assumed errors in the central parts. The results of the new methods were compared to the results of the threshold classification for different lake size classes. The comparison indicates, that the new methods are likely capable of reducing errors observed in the threshold classification for the largest lakes significantly, but no total assessment could be performed because no ground truth data were available. The classifications also show considerable differences between the two years, which might reflect the colder winter of 2017 when compared to 2016. Additionally, three types of features that are thought to cause the differences between the threshold and the other classification approaches were identified. Cracks in the lake ice are thought to be one of them. Other sources are rather wide patches of low backscatter in the SAR images, that could not be related to any phenomenon yet. The third type is dot-like patterns of low backscatter. A time series analysis revealed that these patterns vary over time and tend to intensify in late winter. The presence of locally thin ice could be confirmed by reports from reindeer herders. The patterns also vary between the two years and showed large similarities with optical Sentinel-2 images acquired when the lakes were partly free of snow. It is not clear what these patterns are caused by and only speculations about their origin could be given in this study. However, the patterns seem to be a very interesting subject for future studies.
